# Mean square displacement for a discrete centroid model of cell motion

**DOI:** 10.1371/journal.pone.0261021

**Published:** 2021-12-20

**Authors:** Mary Ellen Rosen, Christopher P. Grant, J. C. Dallon

**Affiliations:** Department of Mathematics, Brigham Young University, Provo, Utah, United States of America; University of Oxford, UNITED KINGDOM

## Abstract

The mean square displacement (MSD) is an important statistical measure on a stochastic process or a trajectory. In this paper we find an approximation to the mean square displacement for a model of cell motion. The model is a discrete-time jump process which approximates a force-based model for cell motion. In cell motion, the mean square displacement not only gives a measure of overall drift, but it is also an indicator of mode of transport. The key to finding the approximation is to find the mean square displacement for a subset of the state space and use it as an approximation for the entire state space. We give some intuition as to why this is an unexpectedly good approximation. A lower bound and upper bound for the mean square displacement are also given. We show that, although the upper bound is far from the computed mean square displacement, in rare cases the large displacements are approached.

## Introduction

One of the characteristics that distinguishes living things from non-living things is motility. On the cellular level, the motility or non-motility of different types of cells can be life-building (e.g., embryogenesis), life-saving (e.g., white blood cells) or life-threatening (e.g., metastasis). A thorough study of cell motility is needed to help understand the underlying mechanisms of motion in order to be able to inhibit or promote cell movement [1]. The mean square displacement (MSD) is often used to analyze particle motion and can be a first indicator of the mechanism of motion in cells. We give a brief introduction to the MSD to give a greater understanding of its origins and applications, including cell motion. It will later be applied to a math model of cell motion.

The MSD is a statistical measure of the average distance a particle travels over time. It can be thought of as a measure of overall drift. For instance, if a particle has a lot of motion within a small radius, its displacement over time may not be a good measure of overall motion, whereas the MSD will capture that. Given a stochastic process *X*(*t*), the MSD is defined as

$$\begin{array}{c} MSD\left(\tau \right)=E[{\left(X\left(\tau \right)-X\left(0\right)\right)}^{2}] \end{array}$$ (1)

(see e.g., [2]). We introduce three common ways to compute the MSD from data. The first two can be applied to any function of time whereas the last method is applied to stochastic processes. The first two use discretized time data (whether from discrete or continuous data), which is true for our application to cell motion and is typically the case when tracking particle motion.

For the first two methods to approximate the MSD we will assume we have particle positions at evenly spaced time intervals. That is, *x*_*i*_ = *x*(*t*_*i*_) for *i* = 0, …*N* where *t*_*i*_ = *t*_0_ + *i*Δ*t*.

The first of the three methods for computing the MSD is useful if the experimental data available is a sufficiently long time trajectory for a single particle, then the time averaged approximation to the MSD (TAMSD), at lag time *τ* = *k*Δ*t* is commonly defined and calculated as follows [3]:

$$\begin{array}{c} TAMSD_{1}\left(\tau \right)=\frac{1}{N-k+1}\sum_{i=0}^{N-k}{\left|x_{i+k}-x_{i}\right|}^{2},\;\;\;\;\;k=1,2,\ldots ,N-1,N. \end{array}$$ (2)

The advantage of Eq (2) is that for small values of *k*, there are many displacements, and the data is well averaged. The disadvantage is that when *k* > 1 there is overlap between the displacements, and successive displacements are usually not independent.

If no overlap is allowed between displacements, then the time averaged approximation to the MSD is computed as

$$\begin{array}{c} TAMSD_{2}\left(\tau \right)=\frac{1}{\left\lfloor (N/k)\right\rfloor }\sum_{i=0}^{\lfloor (N/k)\rfloor -1}{\left|x_{(i+1)k}-x_{ik}\right|}^{2},\;\;\;\;\;k=1,2,\ldots ,N-1,N \end{array}$$ (3)

where ⌊ ⌋ denotes the integer part. This allows displacements to be uncorrelated for calculations, but if *k* is large, it is a poor statistical measure due to fewer sample points. Eqs (2) and (3) are the same when *k* = 1. In the subsequent sections of this paper, we typically assume *k* = 1, and this is the method used when calculating the MSD from simulations (which we refer to as the experimental MSD), since our calculations assume there is independence between successive displacements.

If multiple particles of the same type are being tracked over a short period of time, where each particle is equally weighted, then the ensemble averaged approximation to the MSD (EAMSD) at time *τ* is defined as:

$$EAMSD\left(\tau \right)=\frac{1}{P}\sum_{j=1}^{P}\left(\right|x^{j}\left(\tau \right)-x^{j}\left(0\right){\left|\right)}^{2}$$

where *P* is the number of particles and *x*^*j*^(*τ*) is the location of the *j*-th particle at time *τ*, and *x*^*j*^(0) is the referenced position for the *j*-th particle. When both types of data are available and the system is ergodic (the time average and ensemble average are equivalent for large time) [4], then a simultaneous time and ensemble average is sometimes used, where a time average MSD is computed for each particle and that is then averaged over all the particles. This is especially helpful when lag times are long and improves the statistics [5]. The *EAMSD* is applied to a stochastic process where *x*^*j*^ are different realizations of the stochastic process. In the centroid model which we analyze in this paper, time is discrete so our data takes the form *X*(*ω*)_*i*_ = *X*(*ω*, *i*) and Δ*t* has no meaning (*τ* = *k*). For example if *ω* is specified then *X*(*i*) = *x*^*j*^(*i*). In this setting, the EAMSD is easily recognized as an approximation to the definition of the MSD.

The historical importance of MSD came in the year 1905, when Einstein published his Annus Mirabilis (“extraordinary year”) papers, the second of which contained his research and results on Brownian motion [6]. From his work on the diffusion equation in one dimension he was able to find a linear, time dependent relationship between the MSD and the diffusion coefficient, *D*, which is a measure of the rate that a particle can move through a fluid that is in thermal equilibrium. The relationship is given by *MSD*(*τ*) = 2*Dτ* in one dimension and is extended to *MSD*(*τ*) = 2*dDτ* for a *d*-dimensional system. It was a landmark paper and established the value of statistical mechanics in research. (The continuous time stochastic processes in these applications is approximated by discrete-time processes.) The relationship for MSD was further extended to the viscosity of a purely viscous fluid at thermal equilibrium by research simultaneously developed by both Einstein and Sutherland, although Sutherland’s contributions were only recognized recently [7]. The relationship between the diffusion coefficient and the viscosity *η* of a fluid is given by the Stokes-Einstein-Sutherland relation *D* = *k*_*B*_ *T*/(6*πηR*_*p*_) where *k*_*B*_ is Boltzmann’s constant, *T* is the absolute temperature, and *R*_*p*_ is the radius of a particle, and the particle experiences Stokes drag [6, 8]. Thus, *MSD*(*τ*) = 2*dτk*_*B*_ *T*/(6*πηR*_*p*_). Experimentalists now had a way to calculate the diffusion coefficient. (Its extension to cell motion will be discussed in coming paragraphs.).

Further research has shown that the MSD can be used to determine features of the local rheology of non-Newtonian viscoelastic fluids. The complex shear modulus [5], the dynamic moduli [9], and the creep compliance [10] for these fluids can be found using the MSD. A power law tau dependence between the MSD and tau given by *MSD*(*τ*) = *Aτ*^*α*^ is indicative that a particle is moving by nondiffusive transport when *α* ≠ 1. It also describes diffusion through a viscoelastic medium [11, 12]. The exponent, *α*, is referred to as the MSD scaling exponent, and for physical processes 0 ≤ *α* ≤ 2. When *α* < 1 the process is considered subdiffusive, and for *α* > 1, it is superdiffusive. When the MSD exhibits the relationship *MSD*(*τ*) = 4*Dτ* + (*Vτ*)^2^ with *V* being velocity, the particle exhibits directed motion with diffusive behavior. These different relationships indicate that the MSD, along with the diffusion coefficient, are helpful in revealing the mode of transport, but not all of the mechanisms driving the transport [13].

For living cells, the Stokes-Einstein-Sutherland relation and other equations derived to explain diffusive processes cannot immediately be applied, since living cells use thermal energy and active transport. Under certain conditions, such as active transport inhibition, they are still relevant and can provide information about transport. The time dependent power law is also a useful tool in understanding motion in living cells. Single and two-particle tracking of particles inside a cell have been done on a large number of cell types to find the MSD and hence the MSD scaling exponent [13]. For living cells, if the scaling exponent is in the subdiffusive range, then it may be indicative of a dense intracellular environment and/or there may be numerous reactions and obstacles inside the cell [14]. If the scaling exponent is in the superdiffusive range, then active transport is present [15]. It was also found when tracking whole cells that there is an inverse relationship between the MSD and the stiffness of a cell [16]. This relationship was seen in cancerous cells when the stiffness of the cell decreased as the cell increased in metastatic potential [17]. In summary, the MSD is not only a useful tool to indicate the transport type and mechanics in living cells [13], but in some cases can give information on specific behaviors. Regardless of the application, the MSD is an important statistical measure on a stochastic process.

In this paper we will first discuss the MSD for a simple random walk. We then discuss calculating the MSD for a mathematical model for cell motion. A good estimate for the MSD is found as well as an upper and lower bound for the MSD for this model. We then compare and contrast numerical results found for the simple random walk and our model. Given these methods for estimating the MSD of cell motion with this model, the historical uses of the MSD can be applied to further examine and understand the mechanisms of motion.

## Random walks

Some background in simple random walks is needed in order to better understand the model describing cell motion in the next section, so a brief discussion of random walks and the MSD for random walks is given here. A random walk or drunkard’s walk was first referred to in 1905 in the journal *Nature* in a discussion between Pearson and Rayleigh, demonstrating the theorem, “the most likely place to find a drunken walker is somewhere near his starting point [18].” Since that time, random walk theory has been studied extensively, impacting many important fields, such as random processes, random noise, stochastic equations and spectral analysis. For a more thorough discussion of random walks in biology, see “Random Walk Models in Biology”, by Codling, et.al. [19].

A simple random walk refers to a stochastic process that is the equivalent of a succession of random steps in some finite space or grid. One feature of a random walk is that the jumps are independent. A simple random walk is both time homogeneous (P(X(t)=j|X(0)=a)=P(X(s+t)=j|X(s)=a)) and space homogeneous (P(X(t)=j|X(0)=a)=P(X(t)=j+b|X(0)=a+b)) [20]. Since the process is space homogeneous, we can assume that **X**(0) = **0**, for our purposes. These properties of simple random walks then give that $E\left[{\left\|X(t+\tau )-X(t)\right\|}^{2}\right]=E\left[{\left\|X(\tau )-X(0)\right\|}^{2}\right]=E\left[{\left\|X(\tau )\right\|}^{2}\right]$. Since $\text{Var}\left(\|X\|\right)=E\left[{\left\|X\right\|}^{2}\right]-{\left\|E[X]\right\|}^{2}$, then $E\left[{\left\|X(\tau )-X(0)\right\|}^{2}\right]=\text{Var}\left[\|X(\tau )\|\right]+{\left\|E[X(\tau )]\right\|}^{2}.$ Each **X**(*t*) is the sum of random, independent, identically distributed variables (iids), so **Var**[‖**X**(*τ*)‖] = *τ* **Var**[‖**X**(1)‖] and E[X(τ)]=τE[X(1)].

Then using the definition of the *MSD* as defined in the first section,

$$\begin{array}{l} MSD\left(\tau \right)=E\left[\|X\left(\tau \right)-X\left(0\right){\|}^{2}\right]\\ =\tau \cdot \text{Var}\left[\left\|X\left(1\right)\right\|\right]+\tau^{2}\cdot \|E\left[X\left(1\right)\right]{\|}^{2}. \end{array}$$ (4)

This relation shows that the MSD for a simple random walk is a quadratic function in *τ*. In addition to simple random walks, Eq (4) holds true for any process that is both time and space invariant with **X** being the sum of iids.

Eq (4) is the definition for the MSD used in the next section for our calculations to find the MSD for the model of cell motion. Given the expectation and variance, Eq (4) gives us a way to compute the MSD as a function of *τ*. In a later section, we compare Eq (4) with results from simulations of our model of cell motion (which is not a sum of iids) to see if it has a similar behaviour.

## Finding an estimate for the MSD for a model of cell motion

**Fig 1:**
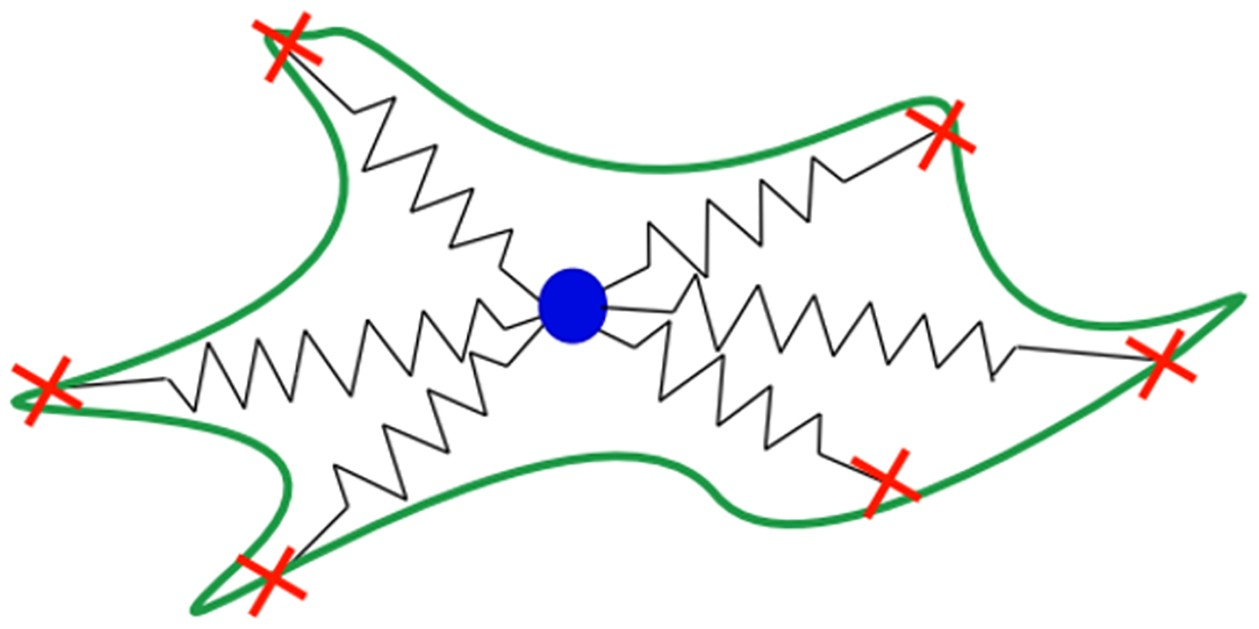
Cell model. This figure from Dallon, et.al. [21] depicts the way the cell is being modeled mathematically. The cell is a center location (nucleus) with attached springs. The other ends of the springs correspond to the different FAs that are attached to the extracellular matrix at “x”.

In a paper by John Dallon, et.al. [21], they introduce a mathematical model of individual cell migration. The model specifies discrete focal adhesion (FA) attachment sites with random switching terms for each site. (A FA is a complex that allows a cell to adhere to the extracellular matrix and is integral to amoeboid cell motion.) The random switching terms determine if a FA is attached or detached. The time a FA remains attached or detached is taken from a given probability distribution. A detached site is reattached at an outreach distance from the present cell center. The outreach is taken from a given probability distribution that specifies the distance and location from the centroid where the FA attaches. Forces exerted on the center of the cell by the different FAs are determined by Hooke’s Law. Using Newton’s second law of motion, and ignoring the acceleration due to the low Reynolds number, all of these forces together with the drag force, which involves velocity, are summed to produce a differential equation model that has the feature of different FAs attaching and detaching randomly and tracks the movement of the cell over time. See Fig 1.

The equation of motion for the cell location is given by

$$\mu x^{\prime }=-\sum_{i=1}^{n}\alpha_{i}\left(\|x-v_{i}\|-\ell_{i}\right)\frac{x-v_{i}}{\left\|x-v_{i}\right\|}\psi_{i}\left(t\right),$$

where **x** is the location of the center of the cell, *α*_*i*_ is the constant for the *i*th spring with resting length *ℓ*_*i*_, **v**_*i*_ is the location of the *i*th FA, *n* is the total number of FAs, and *ψ*_*i*_(*t*) is the state of the *i*th FA at time *t*. If *ψ*_*i*_(*t*) = 0, then the *i*th FA is detached at time *t*, and if *ψ*_*i*_(*t*) = 1, then the *i*th FA is attached at time *t*.

The equation for the location of the *i*th FA **v**_*i*_ when it is going from detached to attached is given by

$$\begin{array}{c} v_{i}\left(t\right)=\underset{y↗a_{p,i}}{\text{lim}}x\left(y\right)+\eta^{p,i}\;\text{for}\;a_{p,i}\leq t<a_{p+1,i}. \end{array}$$

For each *i* the sequence {*a*_*p*,*i*_} of random variables are the times when *ψ*_*i*_ makes the transition from 0 to 1. The vectors ***η***^*p*,*i*^ are independent, identically distributed random vectors with respect to a distribution *ν* on $R^{2}$. (The vector ***η***^*p*,*i*^ is the outreach. The two superscripts denote the time sequence and FA respectively.) Although the equations of motion are independent of the location of the FA when it is detached, for convenience we assume the location does not change until it reattaches.

In a further paper [22], the differential equation model is approximated heuristically by a problem that tracks the centroid of the cell, **c**^*j*^. This new problem is motivated by informally considering the limit of the differential equation model as the cell spring constants become very large. In this limit, the cell nucleus jumps from centroid to centroid. This model is a discrete-time jump process and is the model for which we estimate the MSD.

It is described in the following manner. Let *j* denote the number of binding events (attach or detach events) that have occurred and *n* the number of FAs. The equation describing **c**^*j*^ is

$$0=\sum_{i=1}^{n}\alpha_{i}\left(c^{j}-v_{i}^{j}\right)\psi_{i}^{j}$$

where $v_{i}^{j}$ is the location of the *i*th attachment site at stage *j*, *α*_*i*_ is the spring constant for the *i*th attachment and $\psi_{i}^{j}$ is either 1 if the *i*th attachment site is attached at event *j* or 0 if the *i*th attachment site is detached at event *j*. If the *j*th event is the attachment of **v**_*i*_, then its location is governed by

$$\begin{array}{c} v_{i}^{j}=c^{j-1}+\eta^{j}. \end{array}$$

The vectors ***η***^*j*^ are independent, identically distributed random vectors with respect to a distribution *ν* on $R^{2}$. (For our simulations, the outreach is specified by a length and an angle.).

Each attached FA has a certain probability *p* of changing status, and each detached FA has a probability *rp* of changing status, where *r* > 0. If *k* is the number of attached FAs, then *kp* + (*n* − *k*)*rp* = 1. Thus for given *k*, the probability of going from *k* to *k* + 1 attachments is given by *rp*_*k*_ with

$$\begin{array}{c} p_{k}=\frac{1}{k+(n-k)r} \end{array}$$ (5)

as found in [22]. The probability *p*_*k*_ is the probability of going from *k* to *k* − 1 attachments.

The behavior of the system can be quite complex unless we restrict the initial conditions to be compatible with a steady state distribution of the number of attached FAs. It was previously shown that this distribution is a globally attracting steady state, so if the process is run long enough this is not very restrictive.

More precisely, we restrict the initial conditions to come from a distribution *ρ*, which is a distribution on the Borel sets of the possible cell states, B(X), where

$$X≔\{\left(\left(\psi_{1},\ldots ,\psi_{n}\right),\left(v_{1},\ldots ,v_{n}\right),c\right)\in {\left\{0,1\right\}}^{n}\times {\left(R^{2}\right)}^{n}\times R^{2}:\sum_{i=1}^{n}\psi_{i}\left(v_{i}-c\right)=0\}.$$

(We give *X* the product topology with the discrete topology on {0, 1} and the standard topology on R.) We put a further restriction on *ρ*, such that the probabilities of a projection of *X* onto the number of attachments |*ψ*| associated with any given configuration is consistent with the steady state distribution. This is given by the equation

$$\rho \left(\left(\left(\psi_{1},\ldots ,\psi_{n}\right)\times {\left(R^{2}\right)}^{n}\times R^{2}\right)\cap X\right)=\pi_{\left|\psi \right|}$$

for every (*ψ*_1_, …, *ψ*_*n*_) ∈ {0, 1}^*n*^ with *π*_|*ψ*|_ being the probability of the projected steady state. This steady state was computed in Dallon, et al. [22] and is given by

πk=rk-1(n-1k-1)2(1+r)n-1k[k+(n-k)r], (6)

the probability of being in the projected state of *k* attachments for any configuration with 0 < *k* ≤ *n* and *π*_0_ = 1/(2(1 + *r*)^*n*−1^).

Analysis of the centroid model by the authors in [22] produced an explicit formula for the first moment, given by

Eρ[cj+1-cj]=(1+∑k=1n(rk-1(1-r)(n-1k-1)+rk(nk))r(n-k)(k+r(n-k))(k+1))Eν[η]2(1+r)n-1 (7)

where *ρ* is a probability measure on the Borel sets of the state space as defined above [22].

It is noted that the MSD of the centroid in this setting changes the meaning of *τ* from a time shift to an event shift. We work to determine a similar formula for the MSD of one event shift (*τ* = 1), i.e. $E_{\rho }\left[{\left\|c^{j+1}-c^{j}\right\|}^{2}\right]$ for any *j* ≥ 0.

**Case**: *n* = 1

Consider *n* = 1 with |*ψ*^*j*^| = 0 where |*ψ*^*j*^| is the number of attached sites at time *j*, and compute **c**^*j*+1^ − **c**^*j*^. Since the process is space invariant, then we can assume that **c**^*j*^ = 0. In this case, the difference **c**^*j*+ 1^ − **c**^*j*^ would be the outreach from the centroid on the next step, ***η***^*j*+1^. For |*ψ*^*j*^| = 1, the only possibility for the next event would be going from one attachment to no attachments. (We assume that if all the FAs detach, then the location of the centroid does not move.) In this case, the centroid does not move, so **c**^*j*+1^ − **c**^*j*^ = 0. Those two cases then give the only possible values for the random variables, **c**^*j*+1^ − **c**^*j*^, in the stochastic process when *n* = 1. Thus for *n* = 1, $E_{\rho }\left[{\left\|c^{j+1}-c^{j}\right\|}^{2}\right]=\pi_{0}rp_{0}E_{\nu }\left[{\left\|\eta \right\|}^{2}\right]+\pi_{1}p_{1}E_{\nu }\left[0\right]=\frac{1}{2}r(\frac{1}{r})E_{\nu }\left[{\left\|\eta \right\|}^{2}\right]=\frac{E_{\nu }\left[{\left\|\eta \right\|}^{2}\right]}{2}$

**Case**: *n* = 2

**Fig 2:**
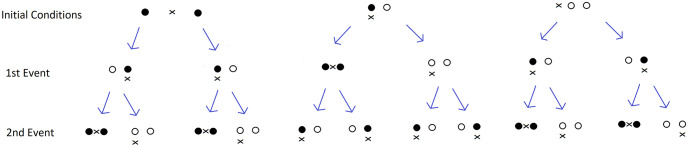
Visualization of the centroid model (n = 2). The left column shows the three possible initial conditions: No attached FAs, one attached FA and 2 attached FAs (in any configuration). The arrows point to possible transitions. Distance is measured vertically. The open dots indicate a detached adhesion site and a black dot represents an attached adhesion site. An “x” indicates the centroid. When the centroid is in the same position as a dot, then it is indicated to the right of the dot.

For *n* = 2, **c**^*j*+1^ − **c**^*j*^ (for any *j* ≥ 1) can be computed for all scenarios of FA attachments/detachments. See Table 1. A visualization for *n* = 2 can be found in Fig 2. Note that the open dots indicate a detached adhesion site and a black dot represents an attached adhesion site. An “x” indicates the centroid.

**Table 1:** Centroid model (n = 2) for *j* ≥ 1. This table shows the probabilities of being in certain states, the probabilities of changing to different states with the number of possibilities for those changes, and the centroid location change.

Probability of State	|*ψ*^*j*^|	|*ψ*^*j*+1^|	Possibilities	c^*j*+1^ − c^*j*^	Probability of Attach/Detach
$\pi_{0}=\frac{1}{2(1+r)}$	0	1	(21)	***η*** ^*j*+1^	$rp_{0}=\frac{1}{2}$
$\pi_{1}=\frac{1}{2}$	1	2	(11)	$\frac{\eta^{j+1}}{2}$	$rp_{1}=\frac{r}{1+r}$
	1	0	(11)	0	$p_{1}=\frac{1}{1+r}$
$\pi_{2}=\frac{r}{2(1+r)}$	2	1	(21)	$\pm \frac{\eta^{j}}{2}$	$p_{2}=\frac{1}{2}$

For *n* = 2, the MSD can be computed by substituting the values of the random variables and associated probabilities found in [22] into Eq (1) with *τ* = 1 and is $\frac{E_{\nu }\left[{\left\|\eta \right\|}^{2}\right]}{2(1+r)}(1+\frac{r}{2})$.

**Case**: *n* > 2

For *n* > 2, the initial condition is a cell that has not yet formed attachments to the substrate, i.e., initially no FAs are attached. In order to find a good estimate for the theoretical MSD, we considered two features of the model: (1) Only one event happens at a time. and (2)The probability that a single FA (focal adhesion) remains attached for a long period of time is small.

**Fig 3:**
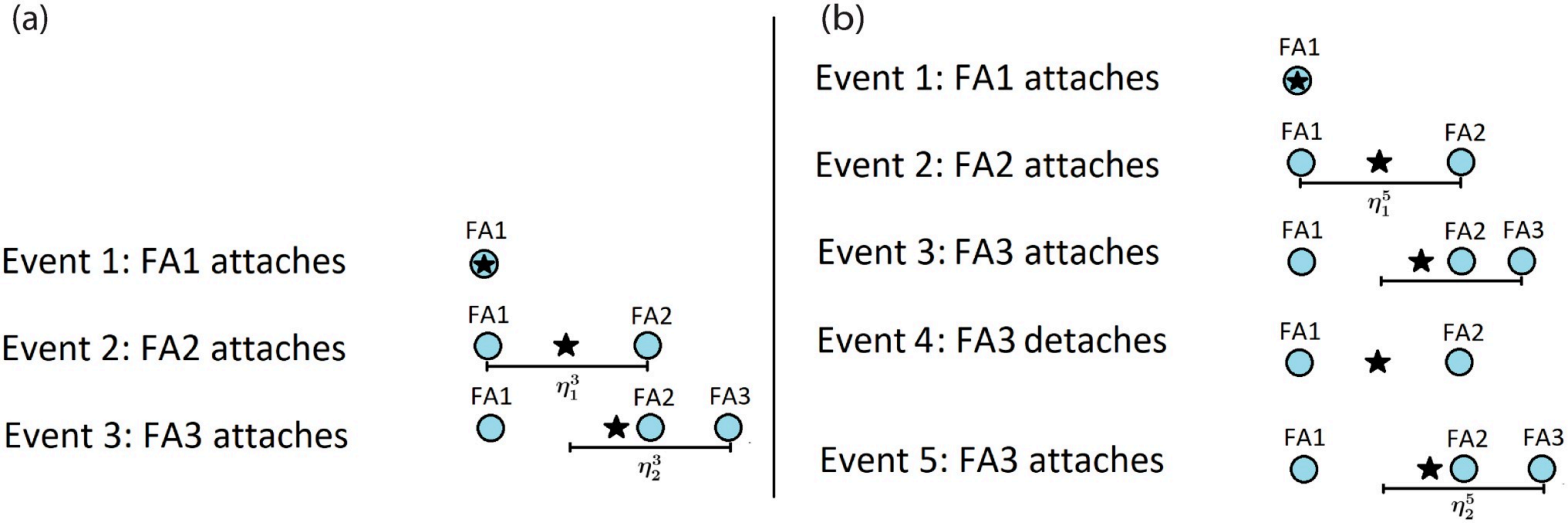
Visualization of sequential attachment configuration with two different histories. For simplicity, the visualization is in one dimension. In panel (a), the first FA (FA1) attaches, denoted by the blue circle. The location of the centroid **c**^1^ is denoted by the star. The next FA (FA2) attaches at distance ***η***_1_ from the centroid. The new centroid is calculated, **c**^2^. The third FA (FA3) attaches at a distance ***η***_2_ from the centroid. A new centroid is calculated, **c**^3^. Panel (b) shows a different history that still has the same sequential configuration as panel (a) because they share a common sequential creation story. The sequential creation story for event 5 has the sequential attachments happening at Event 2 and Event 5.

These two features imply that probabilistically, the FAs will be close together throughout the process. To find a computable estimate for the MSD, we introduce the concept of sequential attachments. By sequential attachments, we mean that for any *k* ≤ *n*, the *k* attachments are sequential if they are in a configuration that can be arrived at by starting with a centroid and no attachments and then attaching one FA at a random outreach (*ν*-distributed) from the centroid. Then the new centroid location is computed and another FA attaches at a random outreach from the centroid. Each new FA attaches in this same way until *k* are attached. The FAs are also considered sequential if they are in the configuration described above whether or not they arrived in that manner. In other words, FAs are in a sequential configuration if they have a sequential creation story. See Fig 3. (Not all attachments are in a sequential configuration. For example, in panel (b) of Fig 3, if at Event 4, FA2 detaches, then the configuration is no longer sequential.) Assuming sequential attachments assures that the FAs are close together and makes it possible to compute the displacement of the centroid when a detach event occurs.

The values for **c**^*j*+1^ − **c**^*j*^ are computed for *n* = 5 as shown in Table 2. The values for an attach event are valid for any configuration in the state space, but the ones for a detach event are only valid if the configuration is a sequential attachment. For the purposes of finding an estimate for the MSD, we assign the full probabilities of the state space to both attach and detach events, even though the random variable for the detach events is only for a sequential configuration. Since the FAs in general are close together, and a sequential configuration achieves this proximity, then assigning the full probability to this case is reasonable.

**Table 2:** Centroid model (n = 5).

Probability of State	|*ψ*^*j*^|	|*ψ*^*j*+1^|	Possibilities	c^*j*+1^ − c^*j*^	Probability of Attach/Detach
$\pi_{0}=\frac{1}{2{\left(1+r\right)}^{4}}$	0	1	(51)	$\eta_{1}^{j+1}$	$rp_{0}=\frac{1}{5}$
$\pi_{1}=\frac{1+4r}{2{\left(1+r\right)}^{4}}$	1	2	(41)	$\frac{\eta_{2}^{j+1}}{2}$	$rp_{1}=\frac{r}{1+4r}$
	1	0	(11)	0	$p_{1}=\frac{1}{1+4r}$
$\pi_{2}=\frac{2r(3r+2)}{2{\left(1+r\right)}^{4}}$	2	3	(31)	$\frac{\eta_{3}^{j+1}}{3}$	$rp_{2}=\frac{r}{2+3r}$
	2	1	(21)	$\pm \frac{\eta_{2}^{j}}{2}$	$p_{2}=\frac{1}{2+3r}$
$\pi_{3}=\frac{2r^{2}\left(2r+3\right)}{2{\left(1+r\right)}^{4}}$	3	4	(21)	$\frac{\eta_{4}^{j+1}}{4}$	$rp_{3}=\frac{r}{3+2r}$
	3	2	(31)	$\frac{1}{2}\left(\frac{\eta_{2}^{j}}{2}+\frac{\eta_{3}^{j}}{3}\right)$ *	$p_{3}=\frac{1}{3+2r}$
				$\frac{1}{2}(\frac{-\eta_{2}^{j}}{2}+\frac{\eta_{3}^{j}}{3})$ *	
				$\frac{-\eta_{3}^{j}}{3}$ *	
$\pi_{4}=\frac{r^{3}\left(r+4\right)}{2{\left(1+r\right)}^{4}}$	4	5	(11)	$\frac{\eta_{5}^{j+1}}{5}$	$rp_{4}=\frac{r}{4+r}$
	4	3	(41)	$\frac{1}{3}(\frac{\eta_{2}^{j}}{2}+\frac{\eta_{3}^{j}}{3}+\frac{\eta_{4}^{j}}{4})$ *	$p_{4}=\frac{1}{4+r}$
				$\frac{1}{3}(-\frac{\eta_{2}^{j}}{2}+\frac{\eta_{3}^{j}}{3}+\frac{\eta_{4}^{j}}{4})$ *	
				$\frac{1}{3}(-\frac{2\eta_{3}^{j}}{3}+\frac{\eta_{4}^{j}}{4})$ *	
				$-\frac{\eta_{4}^{j}}{4}$ *	
$\pi_{5}=\frac{r^{4}}{2{\left(1+r\right)}^{4}}$	5	4	(51)	$\frac{1}{4}\left(\frac{\eta_{2}^{j}}{2}+\frac{\eta_{3}^{j}}{3}+\frac{\eta_{4}^{j}}{4}+\frac{\eta_{5}^{j}}{5}\right)$ *	$p_{5}=\frac{1}{5}$
				$\frac{1}{4}\left(-\frac{\eta_{2}^{j}}{2}+\frac{\eta_{3}^{j}}{3}+\frac{\eta_{4}^{j}}{4}+\frac{\eta_{5}^{j}}{5}\right)$ *	
				$\frac{1}{4}\left(-\frac{2\eta_{3}^{j}}{3}+\frac{\eta_{4}^{j}}{4}+\frac{\eta_{5}^{j}}{5}\right)$ *	
				$\frac{1}{4}(-\frac{3\eta_{4}^{j}}{4}+\frac{\eta_{5}^{j}}{5})$ *	
				$-\frac{\eta_{5}^{j}}{5}$ *	

The superscripts are an event counter, and the subscript on ***η*** for an attach event is the outreach to the *k*th FA and for a detach event is the outreach order in the creation story of the sequential configuration.

*The starred values are only valid for sequential attachments. (For the purposes of finding an estimate for the MSD, the probabilities on the table are for the entire state space even though the random variables for a detach event are only valid for a sequential configuration.).

In general, for *n* total FAs

$$\begin{array}{c} c^{j+1}-c^{j}=\frac{\eta_{k}^{j+1}}{k} \end{array}$$ (8)

when going from |*ψ*^*j*^| = *k* − 1 to |*ψ*^*j*+1^| = *k* attached sites (an attach event) with 1 ≤ *k* ≤ *n*, where the superscripts are an event counter, and the subscript on ***η*** for an attach event is the outreach to the *k*th FA. When going from |*ψ*^*j*^| = *k* to |*ψ*^*j*+1^| = *k* − 1 attached sites (a detach event) with 2 ≤ *k* ≤ *n* then the subscript on ***η*** is the outreach order in the creation story of the sequential configuration. In this case, there are (k1) possibilities for **c**^*j*+1^ − **c**^*j*^. For *ℓ* = 0 to *k* − 2, the *ℓ*th possibility is

$$\begin{array}{c} \frac{1}{k-1}\sum_{i=0}^{k-(\ell +2)}\frac{\eta_{k-i}^{j}}{k-i}-\frac{\ell \eta_{\ell +1}^{j}}{\left(\ell +1\right)}. \end{array}$$ (9)

The last possibility is

$$\begin{array}{c} -\frac{\eta_{k}^{j}}{k}. \end{array}$$ (10)

Each of these possibilities, corresponds to a particular site in the creation story detaching on event *j* + 1.

If we consider each configuration of **c**^**j**+**1**^ − **c**^**j**^, where the number of attachments is known as is the nature of the next event (attach or detach), and consider the possible values of that difference as random variables which depend only on the distribution *ν*, then we can determine expectations. By using Eqs (8), (9) and (10), we can determine expectations with respect to *ν* that contribute to an MSD estimate of the full state space.

**Configuration attach**:

We find $E_{\nu }\left[{\left\|c^{j+1}-c^{j}\right\|}^{2}\right]$ for any number of attachments, *k*, with the next event being an attachment by using Eq (8). Thus, for |*ψ*^*j*^| = *k* − 1 and |*ψ*^*j*+1^| = *k* and 1 ≤ *k* ≤ *n* − 1 then

$$\begin{array}{c} E_{\nu }[{\left\|c^{j+1}-c^{j}\right\|}^{2}]=\frac{E_{\nu }\left[{\left\|\eta^{j+1}\right\|}^{2}\right]}{k^{2}}=\frac{E_{\nu }\left[{\left\|\eta \right\|}^{2}\right]}{k^{2}} \end{array}$$ (11)

where the norm is defined in terms of the inner product.

**Configuration detach (assuming sequential configuration)**:

Similarly, we find $E_{\nu }\left[{\left\|c^{j+1}-c^{j}\right\|}^{2}\right]$ for any number of attachments *k* with the next event being a detachment using Eqs (9) and (10). Thus, for |*ψ*^*j*^| = *k* and |*ψ*^*j*+1^| = *k* − 1, with 2 ≤ *k* ≤ *n* (**c**^**j**+**1**^ − **c**^**j**^ = 0 when *k* = 1), then

$$\begin{array}{c} E_{\nu }[{\left\|c^{j+1}-c^{j}\right\|}^{2}]=\frac{1}{{\left(k-1\right)}^{2}}\sum_{i=1}^{k-1}\frac{iE_{\nu }\left[{\left\|\eta \right\|}^{2}\right]}{i+1}. \end{array}$$ (12)

Using the expectations found in Eqs (11) and (12), we derive an estimate for the MSD. For *n* > 1 adhesion sites the estimated theoretical MSD with respect to the initial distribution, **ρ**, that is compatible with the projected steady state found in [22], assuming only a sequential configuration for a detach event with a full state space probability for all events, is given by

MSD(1)=Eρ[‖cj+1-cj‖2]≈Eν[‖η‖2]2(1+r)n-1(1+∑k=1n-1(n-1k)rk(k+1)2+(n-1k)rk(k+1)(k2)∑i=1kii+1). (13)

To find this estimate for the MSD, which we will refer to as AMSD (approximate MSD), we first find the contribution to the expectation due to the attachment events. Eq (11) is multiplied by *π*_*k*_ and by *rp*_*k*_ (the probability of going from *k* to *k* + 1 attachments) and by the number of possibilities *n* − *k*, for 1 ≤ *k* ≤ *n* − 1, given by $\pi_{k}rp_{k}\left(n-k\right)\frac{E_{\nu }\left[{\left\|\eta \right\|}^{2}\right]}{k^{2}}$. Summing these products over *k* gives the first summation, or second term, in Eq (13) with $\frac{E_{\nu }\left[{\left\|\eta \right\|}^{2}\right]}{2{\left(1+r\right)}^{n-1}}$ being factored out from all terms. The first term (“1”) is when *k* = 0. In order to find an estimate for the detach events, we multiply Eq (12), the equation for sequential attachments, by *π*_*k*_ and *p*_*k*_ (the full probability of going from *k* to *k* − 1 attachments) with 1 ≤ *k* ≤ *n*, given by $\pi_{k}p_{k}\frac{1}{{\left(k-1\right)}^{2}}\sum_{i=1}^{k-1}\frac{iE_{\nu }\left[{\left\|\eta \right\|}^{2}\right]}{i+1}$. Summing over all *k*, with an appropriate change of indices yields the third term in Eq (13). In summary, the first term is for the attachment event when *k* = 0, the second term is the sum over all other attachment events and the third term is the sum over detachment events.

Some numerical simulations were conducted to see how closely this formula compares to the experimental MSD (for the full state space—not just sequential attachments), where we assume that *τ* = 1. For 10,000,000 iterations and fixed *r*, the MSD was computed and compared to the number of FAs.

The numerical simulation to determine the experimental MSD begins with the location of the FAs in a circle equally spaced around the origin at a random distance from 0 to 10 with all FAs attached. It proceeds as follows:

1. Generate a number from the standard uniform distribution.
2. If this number is less than *r* * *p* * (number of detached FAs) where *p* = 1/(|*ψ*| + (*n* − |*ψ*|)*r*), then the event is an attachment. Using MATLAB’s random number generator, a random detached FA is selected and its length and angle of outreach is chosen from a random distribution, and it is attached at the chosen length and angle from the present centroid.
3. If #2 is not true, then the event is a detachment. A random attached FA is selected and detached.
4. The new location of the centroid is computed.
5. The location of the centroid is not recorded until a preset amount of events have happened, the burn-in period. (This is done to“wash out” the initial conditions.)
6. The simulation continues until the specified number of events has happened.
7. The data file of the centroid locations at each event is then used to compute the *MSD*(1).

**Fig 4:**
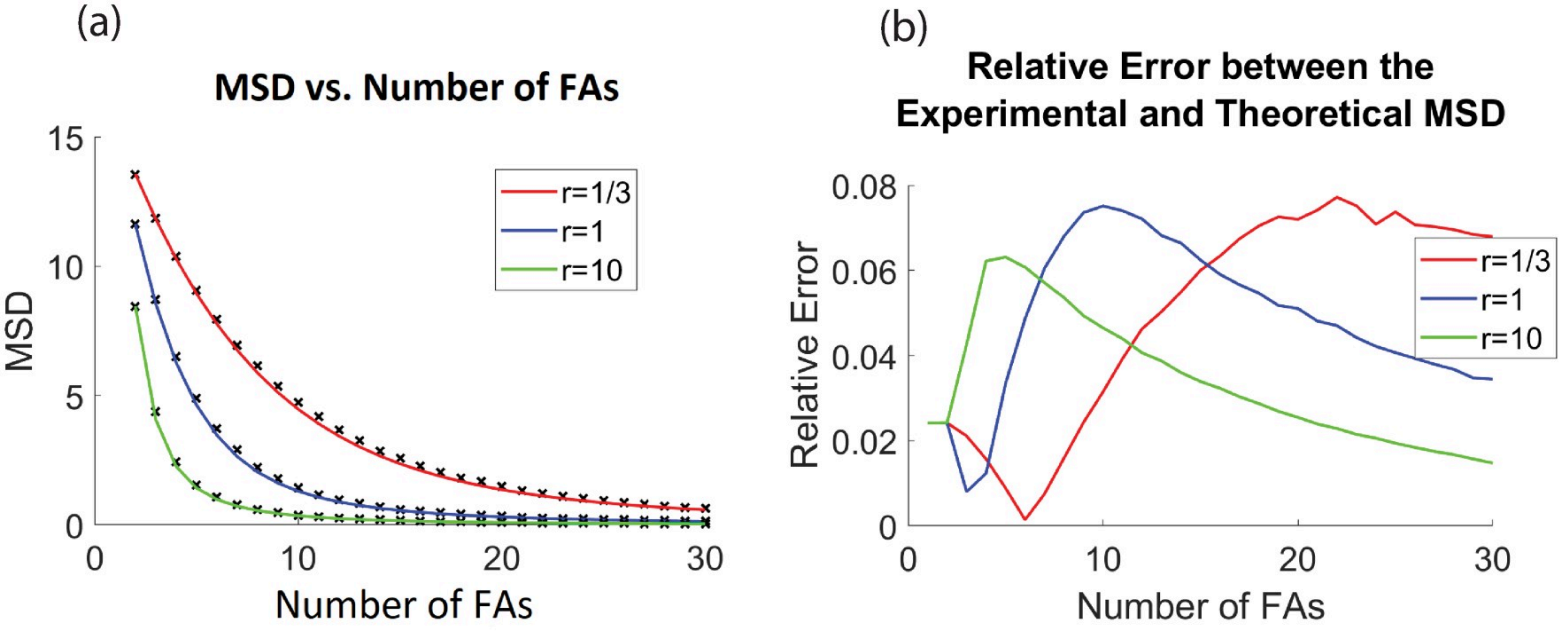
Comparison between the experimental MSD and the estimated theoretical MSD. For the left panel, the experimental MSD (*TAMSD*_2_) is computed from a simulated trajectory of 10,000,000 points and is marked with a black “x” for different values of FAs. It is compared to the AMSD found in Eq (13), given by the solid lines. The right panel shows the relative error between *TAMSD*_2_ and AMSD for different values of *r*. For this simulation and all reported simulations the angle of ***η*** (the outreach vector) is from -30 to 30 degrees, and the length of ***η*** is from 0 to 10. The centroid model is a space and time homogeneous process, thus *TAMSD*_2_ ≈ *MSD*.

The graph of the AMSD from Eq (13) was also computed for fixed *r* and number of FAs and was juxtaposed on the same graph, (see Fig 4). As seen from the graph, Eq (13), is a good estimate for the MSD.

### Lower bound

We now find a lower bound for the MSD. The results will help us understand why the AMSD is such a good approximation. We discuss the insights gained in the section entitled “Discussion on the MSD Estimate”.

In order to find a lower bound for the MSD, given *n* total FAs, we used the random variable values found in Eqs (8), (9) and (10), and for values that are unknown we used 0. For random variable values from Eqs (9) and (10) (a detach event) we used the probability of being in a sequential configuration when the creation story and the actual history coincide. The probability of starting with no attachments is *π*_0_. The probability of attaching one FA is *rp*_0_ multiplied by the number of possibilities of FAs to attach, which is *n*. The probability of attaching another FA is *rp*_1_ multiplied by the number of possibilities, *n* − 1. We continue until we attach the *k*th FA, which has probability *rp*_*k*−1_(*k* − 1). Multiplying all of these probabilities together and then multiplying by *p*_*k*_(*k*) (the probability of being in the state of *k* attachments and detaching one of them) gives the probability of being in this particular sequential configuration of *k* attachments and then detaching one of the FAs. Thus, given *n* FAs, the probability of being in this particular sequential state of *k* attachments and then detaching one of them is

$$\begin{array}{c} P_{k}^{d}\left(r\right)=\pi_{0}\left(rp_{0}n\right)\left(rp_{1}\left(n-1\right)\right)\ldots \left(rp_{k-1}\left(n-\left(k-1\right)\right)\right)\left(p_{k}k\right)\\ =\pi_{0}kr^{k}p_{0}p_{1}\ldots p_{k}(\frac{n!}{(n-k)!})\\ =\frac{1}{2{\left(1+r\right)}^{n-1}}(\frac{r}{nr})(\frac{r}{1+(n-1)r)})\ldots \\ \left(\frac{r}{(k-1)+(n-(k-1))r})(\frac{k}{k+(n-k)r})(\frac{n!}{(n-k)!}\right) \end{array}$$ (14)

where 1 ≤ *k* ≤ *n*. Using these adjusted probabilities for the detach event random variables, we can obtain a lower bound (LB) for the MSD, and it is given by

LB=Eν[‖η‖2]2(1+r)n-1(1+∑k=1n-1((n-1k)rk(k+1)2+rk(k+1)(∏i=1k+11i+(n-i)r)(nk)k!1k2∑j=1kjj+1)). (15)

**Fig 5:**
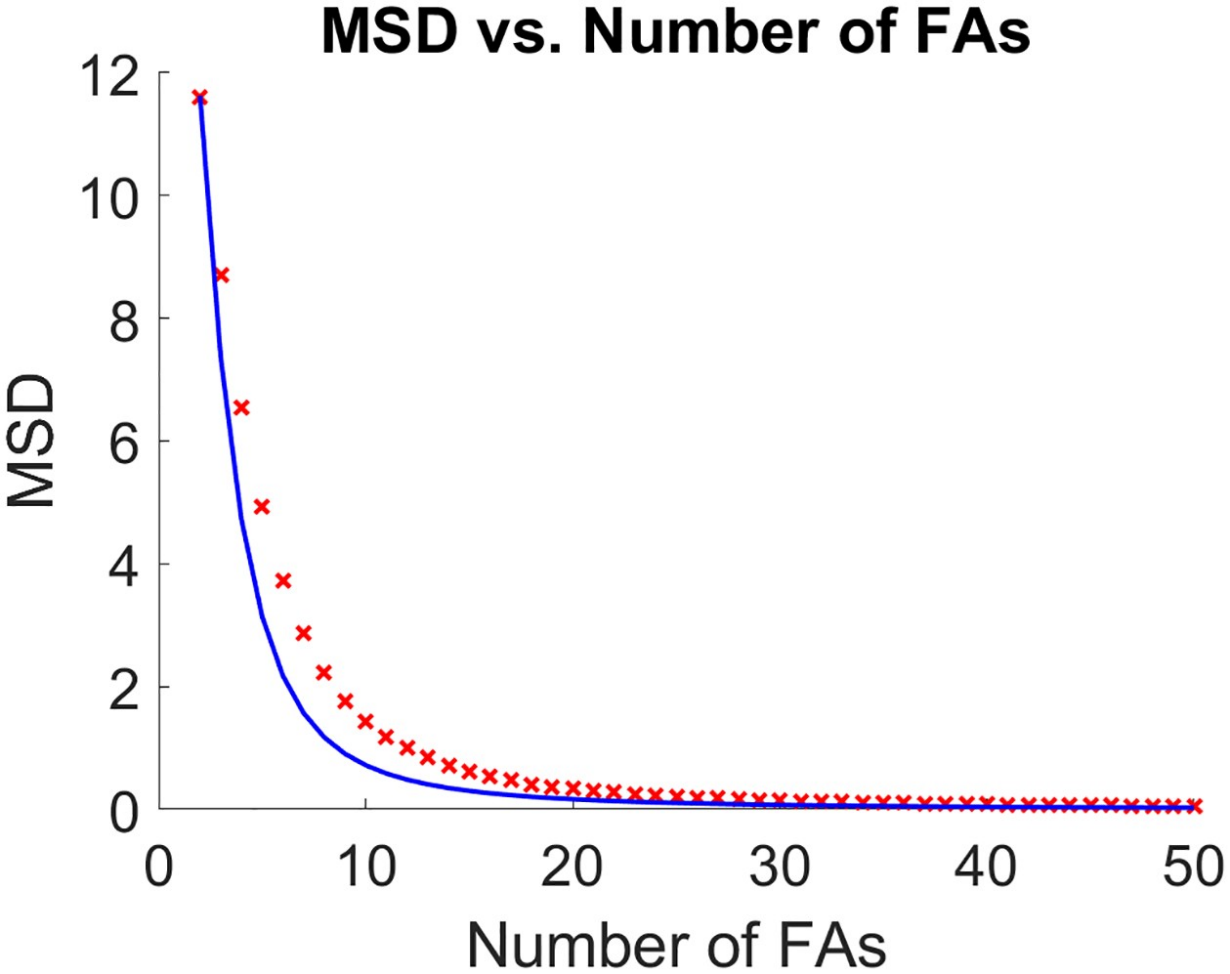
Lower bound for the MSD. Experimental MSD (red x’s) compared against the lower bound for the MSD (blue line) found in Eq (15). For this graph, a trajectory of 10,000 data points was used for the experimental MSD with r = 1.

The first two terms are the same as in Eq (13). The third term is found by using the expectations for a detachment event (assuming sequential configuration) computed in Eq (14), but using the probabilities from Eq (14). A graph of how it compares to the experimental MSD and estimated theoretical MSD can be seen in Fig 5.

### Upper bound

In this section, we seek to find an upper bound for the MSD. In order to find a tight upper bound, it would require an extensive partitioning of the state space, and the possibilities are too numerous to be practicable. Instead, we find a worst-case scenario for the centroid displacement and show that it can be attained, but is highly improbable. Although our upper bound is not ideal, the methods to obtain it are instructive, and at least the existence of an upper bound is proved.

To postulate on the maximum value for ‖**c**^**j**+**1**^ − **c**^**j**^‖, when event *j* + 1 is a detachment, we describe a “worst-case scenario” event. We start with our initial condition assumption of the centroid at the origin with no attachments. Assume the first FA, *v*_1_, attaches at the origin. For simplicity and to obtain a maximum combined outreach, we assume all incremental outreaches occur in one dimension in the positive direction. The next FA, *v*_2_, attaches at a maximum outreach, *η*_*max*_, from the origin. Let each subsequent outreach be at a maximum outreach from the previously attached FA until all *n* FAs are attached, the location of the *i*th FA given by *v*_*i*_, with *v*_1_ = 0 and *v*_*n*_ = *η*_*max*_(*n* − 1). (This is a maximum outreach scenario that is more than the actual model, since the outreach in the model for each new attaching FA is from the centroid.) By fixing *v*_1_ at 0 for all events up through *j*, and allowing *v*_*d*_ to detach (*v*_*d*_ ∈ {*v*_*i*_|1 ≤ *i* ≤ *n*}) for the event *j* + 1 (*j* > *n*) we can find an upper bound for any ‖**c**^**j**+**1**^ − **c**^**j**^‖.

$$\begin{array}{c} \|c^{j+1}-c^{j}\|=\frac{\sum_{i=1}^{n}v_{i}-v_{d}}{n-1}-\frac{\sum_{i=1}^{n}v_{i}}{n}=\frac{\sum_{i=2}^{n}v_{i}-nv_{d}}{n(n-1)}\\ \;\;\leq \frac{\eta_{max}\left(n-1\right)\left(n-1\right)}{n(n-1)}=\frac{\eta_{max}\left(n-1\right)}{n}. \end{array}$$ (16)

where the values after the inequality come from taking the max value for all *v*_*i*_ and taking the minimum value of 0 for *v*_*d*_.

In general, for *k* attachments (1 ≤ *k* ≤ *n*) we find an upper bound for the displacement by using the upper bound configuration found in Eq (16), i.e. all nonzero FAs are *η*_*max*_(*n* − 1) units away from the origin. So for *j* > *n*

$$\begin{array}{c} \|c^{j+1}-c^{j}\|\leq \frac{\eta_{max}\left(n-1\right)\left(k-1\right)}{k-1}-\frac{\eta_{max}\left(n-1\right)\left(k-1\right)}{k}=\frac{\eta_{max}\left(n-1\right)}{k}. \end{array}$$ (17)

We now show analytically that the maximum displacement bound found in Eq (16) can be achieved in the limit. Given *n* FAs, there is a linear recurrence relation for the location of the next FA, given the location of the previous *n* − 1 FAs, where each *x*_*i*_ is the location of a FA and *t* ≥ *n*.

$$x_{t}=\frac{\left(x_{t-1}+x_{t-2}+\ldots +x_{t-n+2}+x_{1}\right)}{n-1}+\eta_{max}$$

or

$$\begin{array}{c} x_{t}=\frac{\left(x_{t-1}+x_{t-2}+\ldots +x_{t-n+2}\right)}{n-1}+\eta_{max} \end{array}$$ (18)

since *x*_1_ = 0.

The steady state of this equation is found by setting all values of *x* to x* and solving for x*. The steady state is then x* = *η*_*max*_(*n* − 1). In order to find if this is an attracting steady state, let *y*_*t*_ = *x*_*t*_ − x*, and Eq (18) becomes

$$\begin{array}{c} y_{t}=\frac{\left(y_{t-1}+y_{t-2}+\ldots +y_{t-n+2}\right)}{n-1}. \end{array}$$ (19)

The characteristic equation for this recurrence relation is

$$\begin{array}{c} \left(n-1\right)\lambda^{n-2}=\lambda^{n-3}+\ldots +\lambda +1. \end{array}$$ (20)

For ease of computation consider the equivalent system

$$\left(k+1\right)\lambda^{k}=\lambda^{k-1}+\ldots \lambda +1$$

where *k* + 1 = *n* − 1. Thus the characteristic polynomial is $\lambda^{k}-\frac{\lambda^{k-1}}{k+1}-\ldots -\frac{\lambda }{k+1}-\frac{1}{k+1}$.

By Descartes rule of signs, we know that the polynomial has exactly one positive real root. Since one and negative one are not roots of the polynomial, the upper and lower bound theorem for real roots of polynomials says that all of the real roots lie between negative one and one. In particular, the unique positive root, call it *ζ*, must be between 0 and 1, i.e. 0 < *ζ* < 1. Further analysis shows that $x^{k}-\frac{x^{k-1}}{k+1}-\ldots -\frac{x}{k+1}-\frac{1}{k+1}<0$ or $x^{k}\leq \frac{x^{k-1}}{k+1}+\ldots +\frac{x}{k+1}+\frac{1}{k+1}$ for all values of 0 ≤ *x* < *ζ* and $x^{k}-\frac{x^{k-1}}{k+1}-\ldots -\frac{x}{k+1}-\frac{1}{k+1}\geq 0$ for *x* ≥ *ζ*. Let *z*_0_ be a complex root of the characteristic polynomial, then $z_{0}^{k}-\frac{z_{0}^{k-1}}{k+1}-\ldots -\frac{z_{0}}{k+1}-\frac{1}{k+1}=0$. Using the triangle inequality, then $|z_{0}{|}^{k}\leq \frac{|z_{0}{|}^{k-1}}{k+1}+\ldots +\frac{|z_{0}|}{k+1}+\frac{1}{k+1}$. This implies that 0 < |*z*_0_|<*ζ* < 1. Since *z*_0_ was arbitrary, then all of the complex roots of the characteristic polynomial have modulus less than one. Therefore, all roots of the characteristic polynomial lie within the unit circle in the complex plane, showing that the steady state, x* = *η*_*max*_(*n* − 1) is attracting, and the system will converge to it, since it is the only steady state. As the system approaches the steady state, then the displacement is maximal, and by extending to higher dimensions is given by,

$$\begin{array}{c} \underset{{\text{lim}}_{j\to \infty }}{\left\|c^{j+1}-c^{j}\right\|}=\frac{\eta_{max}\left(n-1\right)\left(n-1\right)}{n-1}-\frac{\eta_{max}\left(n-1\right)\left(n-1\right)}{n}=\frac{\eta_{max}\left(n-1\right)}{n} \end{array}$$ (21)

for *n* total FAs, which is the value seen in Eq (16).

**Fig 6:**
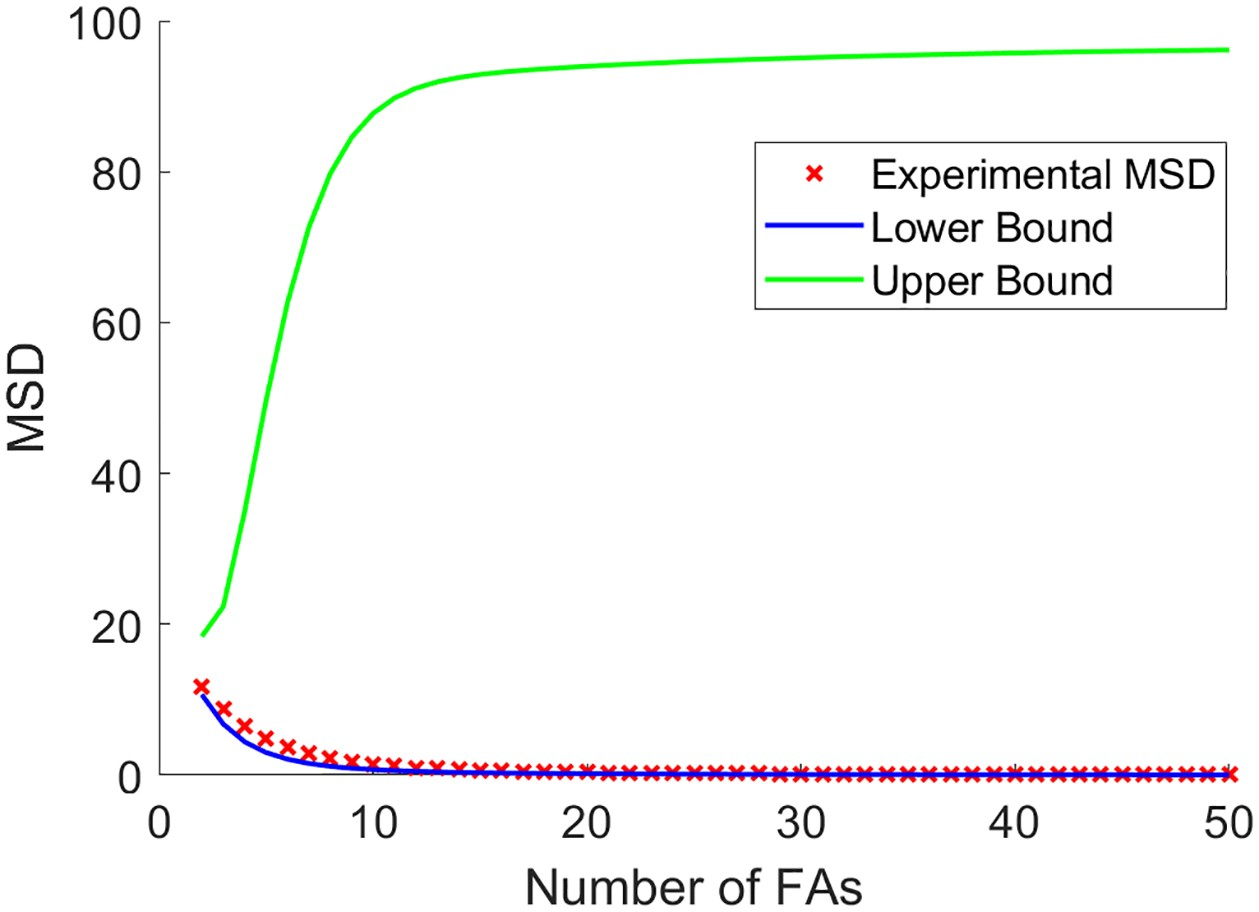
Upper bound for the MSD. An upper bound for the MSD is found using the values for the centroid displacement found in Eq (17). A trajectory of 100,000 data points with *r* = 1 was used to approximate the MSD.

Since the upper bound of the displacement found in Eq (16) can be obtained in the limit (Eq (21)), we now use the results found in Eqs (16) and (17) to find an upper bound for the MSD. We partition the state space into three parts: {$F_{k}^{a}\}$, {${\tilde{F}}_{k}^{d}$} and {$F_{k}^{d}\}$. Each $F_{k}^{a}$, 0 ≤ *k* ≤ *n* − 1, represents arriving to a state of *k* attachments from any configuration and then attaching. Each ${\tilde{F}}_{k}^{d}$, 1 ≤ *k* ≤ *n* represents arriving to the state of *k* attachments from a sequential configuration and then detaching. Each $F_{k}^{d}$, 1 ≤ *k* ≤ *n*, represents arriving to a state of *k* attachments from a non-sequential configuration and then detaching. We use the known values and associated probabilities for $F_{k}^{a}$, and we use the RV values in Eqs (9) and (10) with probabilities from (14) for ${\tilde{F}}_{k}^{d}$ in the computation of the MSD upper bound. We use the results from Eq (17), as a RV upper bound for the event of arriving at *k* attachments from a non-sequential configuration. For the upper bound for the probabilities in this case, we use $k\pi_{k}p_{k}-\pi_{0}kr^{k}p_{0}p_{1}\ldots p_{k}(\frac{n!}{(n-k)!})$ (sequential probability from (14) subtracted from the probability of being in a state of k attachments and then detaching). The resultant upper bound can be seen in Fig 6. For {$F_{k}^{d}\}$, since we use a rare event for the upper bound of the displacement (one FA staying attached for a long time), and multiply it by a large probability, then this is the best estimate for an upper bound that can be found without partitioning the space into the many, many ways that the FAs can arrive at a state of *k* attachments and then have one FA detach.

### MSD as a function of *τ*

Thus far we have considered *MSD*(1) for the centroid model. We now turn our attention to *MSD*(*τ*) where *τ* is an integer and represents the number of binding events between the centroid locations being considered. As mentioned earlier, for a random walk, the shape of the *MSD* curve can reveal if the process is purely diffusive (linear) or has a directed component (quadratic) as can be seen in Eq (4). Eq (4) is valid for more than random walks. It is valid for any process that is both time and space invariant and is the sum of iids. The centroid process we are modeling is both time and space invariant, but as Table 1 indicates, the location of the centroid is not a sum of iids. Since the state space is the location of the centroid and does not include the number of attached FAs, the random variables, **c**^*j*+1^ − **c**^*j*^, for different values of *j*, are not independent. For example, if there are 2 FAs and given some nonzero value for the random variable, **c**^*j*+1^ − **c**^*j*^, within an interval that would satisfy the state of going from none attached to one attached, or from two attached to one attached, or from one attached to two attached, then that probability would be greater than if it was conditioned on the previous random variable being 0.

**Fig 7:**
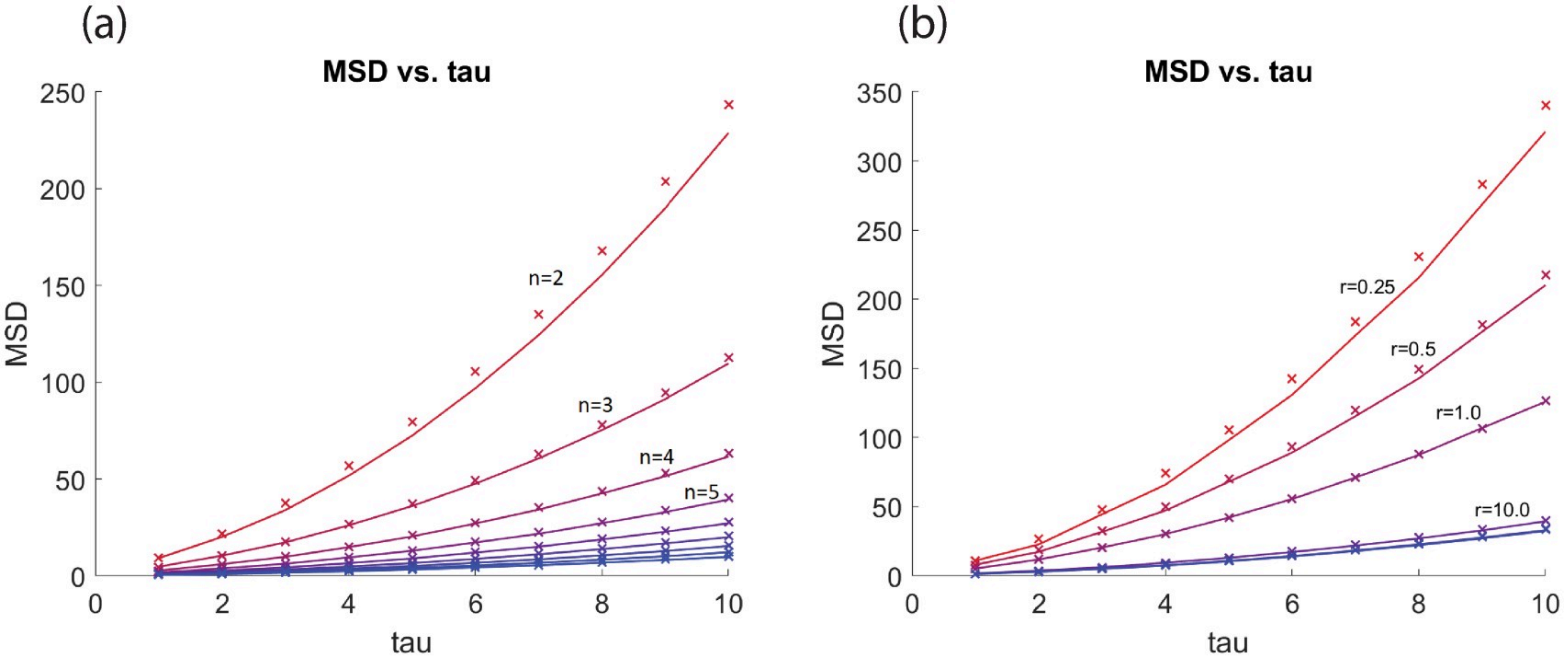
MSD as a function of tau. Panel (a) visualizes the numerical (*TAMSD*_2_) and theoretical (AMSD) results of the MSD versus tau for different values of *n* (2–10), the total number of FAs, and *r* = 10. Panel (b) shows simulations where *r* varies from 0.25, 0.5, 1, 10, 50, and 100 with *n* = 5. The lines represent the relationship between tau and *TAMSD*_2_. The “x” uses the AMSD (Eq (13)) and expectation (Eq (7)) to compute the MSD in Eq (4) for different values of tau. There were 1,000,000 iterations.

Numerical simulations were conducted to see how Eq (4) compares with the experimental MSD as a function of *τ*. The x’s in Fig 7 are calculated using Eqs (4), (13) and (7) in the following way. Since our process is space and time homogeneous we assume *j* = 0 and **c**^0^ = (0, 0) to give

$$E_{\rho }\left[c^{j+1}-c^{j}\right]=E_{\rho }\left[c^{1}-c^{0}\right]=E_{\rho }\left[c^{1}\right].$$

This relates Eq (7) to the expectation term in Eq (4). Furthermore,

$$MSD\left(1\right)=E_{\rho }\left[{\left\|c^{1}-c^{0}\right\|}^{2}\right]={\text{Var}}_{\rho }\left[\|c^{1}\|\right]+{\left\|E_{\rho }\left[c^{1}\right]\right\|}^{2}.$$

Thus using *AMSD* from Eq (13) and the expectation found in Eq (7) to compute the variance and expectation,

τ·Varρ[‖c1‖]+τ2·‖Eρ[c1]‖2≈τ(AMSD(1)-‖Eρ[c1]‖2)+τ2‖Eρ[c1]‖2=τEν[‖η‖2]2(1+r)n-1(1+∑k=1n-1(n-1k)rk(k+1)2+(n-1k)rk(k+1)(k2)∑i=1kii+1)+(τ2-τ)‖Eν[η]‖24(1+r)2(n-1)(1+∑k=1n(rk-1(1-r)(n-1k-1)+rk(nk))r(n-k)(k+r(n-k))(k+1))2.

This gives an estimate of the MSD as a function of *τ* as indicated by Eq (4) with **c** being the random variable. In Fig 7 panel (a) the different curves represent different values of *n* the total number of FAs and panel (b) shows curves where *r*, the propensity to attach, varies.

The graphs reveal that adhesiveness of the cell may be inferred by examining how quickly the MSD increases with respect to *τ*. As the number of FAs increase (or the adhesive ability of the cell), the MSD curve flattens. The same is true for the propensity of FAs to attach. As the value of *r* increases the MSD curve flattens. This work shows that additional information about the cell and its motility can be gained by examining the MSD.

### Discussion on the MSD estimate

In this section we give an explanation as to why our estimate for the MSD is so close to the MSD calculated from simulations. In the section on the lower bound, we found the probability for being in a sequential configuration of *k* attachments when the history and the creation story coincide denoted by $P_{k}^{d}\left(r\right)$ and given in Eq (14). For each *k*, 1 ≤ *k* ≤ *n*, $\underset{r\to \infty }{\text{lim}}P_{k}^{d}\left(r\right)=0$. The total probability of being in this particular sequential configuration for any number of attachments and then detaching is the sum over all *k* of $P_{k}^{d}$, so as *r* increases sufficiently, the probability, $\sum_{k=1}^{n}P_{k}^{d}$ decreases (approaching 0). For *k* = 1, $\underset{r\to 0}{\text{lim}}P_{k}^{d}\left(r\right)=.5$, but for 2 ≤ *k* ≤ *n*, $\underset{r\to 0}{\text{lim}}P_{k}^{d}\left(r\right)=0$. Again, the total probability of being in this particular sequential configuration for any number of attachments and then detaching is the sum over all *k* of $P_{k}^{d}$, so as *r* decreases sufficiently, the probability $\sum_{k=1}^{n}P_{k}^{d}$ increases (approaching .5). Because of the bounds on the number of FAs, over a long enough simulation, on average, the probability of being in a state of any number of attachments and then detaching and the probability of being in a state of any number of attachments and then attaching is equal, and is .5. This helped us better understand why Eq (13) is such a good estimate for the MSD. Heuristically, as *r* decreases sufficiently, the number of attachments decreases, and the sequential probability increases, implying that the random variable (RV) values, **c**^*j*+1^ − **c**^*j*^, being used for a detachment event (Eqs (9) and (10)) are closer to the actual values of the RVs. As *r* increases sufficiently, the number of attachments on average approaches the total number of FAs. Because of the initial condition of starting the simulation with no attachments, FAs quickly attach (*r* is large) until most are attached and the system stays in a highly attached state. Because the majority of attachments happened quickly they will be close to a sequential configuration. Thus the RVs being used for a detachment event (Eqs (9) and (11)) are still a good estimate for the MSD. For the “middle” values of *r*, the estimate is not as good, but is still adequate.

## Conclusion

MSD is a measure of the overall drift of a particle and can be a useful tool for understanding cell motion because it also indicates mode of transport. We introduced a mathematical model for cell motion and discussed it in the context of a a centroid model (a discrete-time jump process). We were able to find a good estimate for the theoretical MSD of the centroid model by introducing the concept of a sequential configuration. We found the displacement after an attach event and the displacement after a detach event when in a sequential configuration. Using the displacement for sequential configurations to approximate all detach events we found a good approximation for the MSD with a delay of one event. To further quantify the MSD, we found a lower and upper bound for the MSD. We surmised that the estimate for the MSD had a small relative error because the FA configuration frequently is in a sequential configuration or close to it.

## Supporting information

S1 File (PDF)
