# Supplementary material for: Mean square displacement for a discrete centroid model of cell motion
Source: PLoS One. 2021 Dec 20;16(12):e0261021. doi: 10.1371/journal.pone.0261021 (PMC8687545; doi:10.1371/journal.pone.0261021)
Supplement: S1 File — (PDF) [file pone.0261021.s001.pdf]

1 Experimental MSD Code

1.1 Main Code

```
%This program computes experimental MSD using the time
%trajectory definition. On the same plot,
%it juxtaposes the AMSD. It plots nint versus the MSD.
global average_length;
global icounter;
Relative_error = figure;
MSD_vs_r = figure;
iterationend = 30;
MSD = zeros(1, iterationend);
%nint = zeros(1,iterationend+1);
nintvector = zeros(1, iterationend);
MSD_formula = zeros(1, iterationend);
for m = 1:3
    if m == 1
        r = 10;
    elseif m == 2

        r = 1;
    elseif m == 3
        r = 1 / 3;
    end
    for j = 1:iterationend
        nint = j;
        nintvector(j) = j;
        %nint=10;
        counter = 10000000;
        min_length = 0;
        max_length = 10;
        angle = 60;
        tau = 1;
        average_length = 0;
        icounter = 0;
        final_data = zeros(counter, 2);
        [x, y, site_state, cx, cy] = Initialize(min_length, max_length, nint, angle);
        for i = 1:50
            [cx, cy] = Compute_Centroid(x, y, nint, site_state, cx, cy);
            [x, y, site_state] = Update_Integrins(x, y, nint, site_state,
                min_length, max_length, angle, cx, cy, r);
        end
        for k = 1:counter
            [cx, cy] = Compute_Centroid(x, y, nint, site_state, cx, cy);
            [x, y, site_state] = Update_Integrins(x, y, nint, site_state,
            min_length, max_length, angle, cx, cy, r);
            final_data(k, :) = [cx, cy];
        end

        [MSD(j)] = Compute_MSD(tau, counter, final_data);

        %figure(MSD_vs_r);
        %hold on;
        %plot(r, MSD,'*', 'Color', 'r', 'LineWidth', 1.5, 'DisplayName',
            'Experimental MSD')
        %xlabel('"r"');
        %set(gca, 'FontSize', 14);
        %ylabel('MSD');
        %set(gca, 'FontSize', 14);
        %title('MSD vs. "r"');
        %set(gca,'FontSize', 18);

        sum1 = 0;
        if nint == 1
            MSD_formula(j) = (1 / (2 * 3 * (max_length - min_length)))
```

```
            * (max_length ^ 3 - min_length ^ 3);
            %MSD_formula(j) = (max_length+min_length)^2/(8);
        else
            for ii = 1:nint - 1
                sigma = 0;
                for jj = 1:ii
                    sigma = sigma + jj / (jj + 1);
                end
                sum1 = nchoosek(nint - 1, ii) * (r ^ ii / ((ii + 1) ^ 2))
                * (1 + (sigma * (ii + 1)) / (ii ^ 2)) + sum1;
            end
            %MSD_formula(j) =
            (max_length + min_length)^2*(1+sum1)/(8*(1+r)^(nint-1));
            MSD_formula(j) = (1 / (3 * (max_length - min_length)))
            * (max_length ^ 3 - min_length ^ 3) * (1 + sum1)
            / (2 * (1 + r) ^ (nint - 1));
        end
        %pause;
        %plot(r, MSD_formula,'*', 'Color', 'b', 'LineWidth', 1.5,
            'DisplayName', 'Formula MSD')
        %legend
        %average_length/icounter
    end
    figure(MSD_vs_r);
    hold on;
    plot(nintvector, MSD, 'x', 'Color', 'r', 'LineWidth', 1.5,
        'DisplayName', 'Experimental MSD')
    xlabel('Number of FAs');
    set(gca, 'FontSize', 14);
    ylabel('MSD');
    set(gca, 'FontSize', 14);
    title({'MSD vs. Number of FAs'; ''});
    set(gca, 'FontSize', 18);
    plot(nintvector, MSD_formula, '-', 'Color', 'b', 'LineWidth', 1.5,
        'DisplayName', 'Formula MSD')
    legend('Experimental MSD', 'Theoretical MSD');
    figure(Relative_error);
    hold on;
    difference = abs(MSD_formula - MSD) ./ MSD_formula;
    xlabel('Number of FAs');
    set(gca, 'FontSize', 14);
    ylabel('Relative Error');
    set(gca, 'FontSize', 14);
    title({'Relative Error between the'; 'Experimental and Theoretical MSD'; ''});
    set(gca, 'FontSize', 18);
    if m == 1
        plot(nintvector, difference, '-', 'Color', 'r', 'Linewidth', 1.5);
    elseif m == 2
        plot(nintvector, difference, '-', 'Color', 'b', 'Linewidth', 1.5);
    elseif m == 3
        plot(nintvector, difference, '-', 'Color', 'g', 'Linewidth', 1.5);
        legend('r=10', 'r=1', 'r=1/3');
    end

end
```

1.2 Initialize Integrins

```
%This program is a function that initializes the center and FA sites in the
%centroid model. Input is the minimum and maximum outreach length, the
%number of FAs (nint), and the angle in degrees on either side of zero for the
%outreach (angle). All FAs are initialized as attached.

function[x, y, site_state, cx, cy] =
```

```
Initialize(min_length, max_length, nint, angle)
    cx = 0;
    cy = 0;
    x = zeros(1, nint);
    y = zeros(1, nint);
    site_state = zeros(1, nint);
    for i = 1:nint
        length = min_length + (max_length-min_length)*rand;
        %length = (min_length + max_length) / 2;
        theta = 360 / nint * i * pi / 180;
        %theta=0;
        x(i) = length * cos(theta);
        y(i) = length * sin(theta);
        site_state(i) = 1;
    end
end
```

1.3 Update Integrins

```
%This program is a function that updates the FAs. It determines what
%kind of event happened, and changes the status of an attached or detached site.
%Inputs for the function are: The x and y coordinates of the i-sites (x,y),
%the number of integrins (nint), the site status vector (site_state),
%minimum and max length of outreach, angle of outreach, location of the
%centroid, (c) and the value of "r" as described in "Cell Speed is
%Independent of Force ...", by Dallon, et. al.

function[x, y, site_state] = Update_Integrins(x, y, nint, site_state,
min_length, max_length, angle, cx, cy, r)
    global average_length;
    global icounter;
    psi = sum(site_state);
    p = 1 / (psi + (nint - psi) * r);
    random = rand;
    numdet = nint - psi;
    if random < r * p * (numdet)
        temp = randi([1 numdet], 1, 1);
        idx = find(site_state == 0, temp, 'first');
        which_integrin = idx(end);
        site_state(which_integrin) = 1;
        length = min_length + (max_length - min_length) * rand;
        theta = - angle * pi / 180 + 2 * angle * rand * pi / 180;
        x(which_integrin) = length * cos(theta) + cx;
        y(which_integrin) = length * sin(theta) + cy;
    else

        temp2 = randi([1 psi], 1, 1);
        idx = find(site_state == 1, temp2, 'first');
        which_integrin = idx(end);
        site_state(which_integrin) = 0;
    end
end
```

1.4 Compute Centroid

```
    %This program is a function that computes the centroid. Input given is the
%FA-site location (x,y), the number of FAs (nint), and the site status
%(site_state) which equals one if attached and 0 if not attached.

function[cx, cy] = Compute_Centroid(x, y, nint, site_state, cx, cy)
    sumx = 0;
    sumy = 0;
    psi = 0;
    for i = 1:nint
        if site_state(i) == 1
```

```
            sumx = sumx + x(i);
            sumy = sumy + y(i);
            psi = psi + 1;
        end
    end
    if psi == 0
        cx = cx;
        cy = cy;
    else
        cx = sumx / psi;
        cy = sumy / psi;
    end
```

1.5 Compute MSD

```
    %This program is a function that computes the MSD, given data from the
%centroid model. Input is the number tau, or lag time between points to be
%compared. The variable counter is how many centroids were computed. Also,
%"c" is the location of the centroid

function[MSD] = Compute_MSD(tau, counter, final_data)
    n = 0;
    sum_msd = 0;
    for i = 1:tau:((counter - mod(counter, tau)) - tau)
        %for i=1:counter-tau
        n = n + 1;
        ss(n, 1) = (final_data(i + tau, 1) - final_data(i, 1));
        ss(n, 2) = (final_data(i + tau, 2) - final_data(i, 2));
        tt(n) = (final_data(i + tau, 1) - final_data(i, 1)) ^ 2 +
        (final_data(i + tau, 2) - final_data(i, 2)) ^ 2;
        sum_msd = tt(n) + sum_msd;
    end
    MSD = sum_msd / n;
    MSD_STD = std(tt);
    VAR = sum(var(ss));
    EXP = mean(ss);
    norm_squared = dot(EXP, EXP);
    clear tt;
```
